# Influenza vaccination and risk of atrial fibrillation in patients with gout: A nationwide population-based cohort study

**DOI:** 10.3389/fphar.2022.990713

**Published:** 2022-09-26

**Authors:** Chun-Chao Chen, Chun-Chih Chiu, Nai-Hsuan Chen, Tsung-Yeh Yang, Cheng-Hsin Lin, Yu-Ann Fang, William Jian, Meng-Huan Lei, Hsien-Tang Yeh, Min-Huei Hsu, Wen-Rui Hao, Ju-Chi Liu

**Affiliations:** ^1^ Division of Cardiology, Department of Internal Medicine, Shuang Ho Hospital, Taipei Medical University, Taipei, Taiwan; ^2^ Taipei Heart Institute, Taipei Medical University, Taipei, Taiwan; ^3^ Division of Cardiology, Department of Internal Medicine, School of Medicine, College of Medicine, Taipei Medical University, Taipei, Taiwan; ^4^ Graduate Institute of Medical Sciences, College of Medicine, Taipei Medical University, Taipei, Taiwan; ^5^ Department of General Medicine, Shin Kong Wu Ho-Su Memorial Hospital, Taipei, Taiwan; ^6^ Division of Cardiovascular Surgery, Department of Surgery, Shuang Ho Hospital, Taipei Medical University, Taipei, Taiwan; ^7^ Division of Cardiovascular Surgery, Department of Surgery, School of Medicine, College of Medicine, Taipei Medical University, Taipei, Taiwan; ^8^ Department of Emergency, University Hospitals Cleveland Medical Center, Cleveland, OH, United States; ^9^ Cardiovascular Center, Lo-Hsu Medical Foundation Luodong Poh-Ai Hospital, Yilan, Taiwan; ^10^ Department of Surgery, Lotung Poh-Ai Hospital, Yilan, Taiwan; ^11^ Graduate Institute of Data Science, College of Management, Taipei Medical University, Taipei, Taiwan; ^12^ Department of Neurosurgery, Wan-Fang Hospital, Taipei Medical University, Taipei, Taiwan

**Keywords:** gout, influenza vaccination, arrhythmia, atrial fibrillation, hyperuricemia

## Abstract

**Objective:** Although influenza vaccination reduces the risk of atrial fibrillation (AF), its protective effect in patients with gout remains unclear. The present study aimed to evaluate the protective effect of influenza vaccination in patients with gout.

**Methods:** A total of 26,243 patients with gout, aged 55 and older, were enrolled from the National Health Insurance Research Database (NHIRD) between 1 January 2001, and 31 December 2012. The patients were divided into vaccinated (*n* = 13,201) and unvaccinated groups (*n* = 13,042). After adjusting comorbidities, medications, sociodemographic characteristics, the risk of AF during follow-up period was analyzed.

**Results:** In influenza, non-influenza seasons and all seasons, the risk of AF was significantly lower in vaccinated than in unvaccinated patients (Adjust hazard ratio [aHR]: 0.59, 95% confidence interval [CI]: 0.50–0.68; aHR: 0.50, 95% CI: 0.42–0.63; aHR: 0.55, 95% CI: 0.49–0.62, respectively). In addition, the risk of AF significantly decreased with increased influenza vaccination (aHR: 0.85, 95% CI: 0.69–1.04; aHR: 0.72, 95% CI: 0.60–0.87; aHR: 0.40, 95% CI: 0.33–0.49, after first, 2–3 times, and ≥4 times of vaccination, respectively). Furthermore, sensitivity analysis indicated that the risk of AF significantly decreased after influenza vaccination for patients with different sexes, medication histories, and comorbidities.

**Conclusions:** Influenza vaccination is associated with a lower risk of AF in patients with gout. This potentially protective effect seems to depend on the dose administered.

## Introduction

Gout is a common form of inflammatory arthritis that is associated with a certain degree of disease burden (Smith et al., 2014; Dehlin et al., 2020). Nearly one in 16 individuals in Taiwan have gout (Kuo et al., 2015). The prevalence of gout in Taiwan is higher than in other countries such as the United Kingdom (2.49%) and the United States (3.9%) (Kuo et al., 2015). Gout results from uric acid deposition; the uric acid links not only to acute or chronic arthritis but also cardiovascular disease (Kim et al., 2016; Singh and Cleveland, 2018; Dehlin et al., 2020; Khan et al., 2021). Past studies demonstrated that the prevalence of atrial fibrillation (AF) and cardiovascular mortality is higher in patients with gout than in those without (Kim et al., 2016; Singh and Cleveland, 2018; Khan et al., 2021).

AF is a common form of cardiac arrhythmia (Chugh et al., 2014). With the growing population of older adults, the prevalence and severity of AF have increased (Savickas et al., 2020). Various factors are related to the incidence of AF, such as hypertension, diabetes, valvular heart disease, and cardiomyopathy (Staerk et al., 2018). Several studies have reported a strong association between AF and gout (Kuo et al., 2016a; Singh and Cleveland, 2018; Khan et al., 2021). In patients with gout, the risk of AF is considerably higher than in those without (Singh and Cleveland, 2018; Khan et al., 2021). Although both AF and gout have some common risk factors, their exact mechanisms remain unclear. This may be due to the role of chronic inflammation, oxidative stress, and uric acid in atrial remodeling processes (Dalbeth and Haskard, 2005; Kuo et al., 2016a).

Influenza is a highly contagious infection and a critical cause of death among senior citizens. Every year, influenza infections result in the death of over 20,000 individuals worldwide, leading to an economic burden (Fang et al., 2016; Smetana et al., 2018). In particular, among patients with chronic diseases, the morbidity and mortality rates associated with influenza infection are high. Besides, previous reports pointed out that influenza may play an important role to trigger or exacerbate the cardiovascular events, including the increased risk of AF (Chang et al., 2016; Pérez-Rubio et al., 2021).

Previous studies have highlighted that influenza vaccination reduces the risks of flu infection, hospitalization, and severe illness (Rondy et al., 2017). Influenza vaccination may also reduce the risks of major adverse cardiovascular events (risk ratio = 0.64, 95% confidence interval [CI] = 0.48–0.86) and even the development of AF among the general population (odds ratio = 0.88, *p* < 0.01) (Phrommintikul et al., 2011; Udell et al., 2013; Chang et al., 2016). Some direct or indirect mechanisms had been proposed to explain the possible protective effect of flu-vaccination on decreasing cardiovascular events (Pérez-Rubio et al., 2021). However, the protective effect of influenza vaccination to decrease risk of AF among patients with gout remained unclear. Thus, in this nationwide population based observational study, the association between the risk of AF and influenza vaccination among patients with gout was investigated.

## Methods

In 1995, Taiwan’s National Health Insurance (NHI) program was established to provide the residents of Taiwan with comprehensive health insurance. This program currently covers more than 98% of the population in Taiwan (Luo et al., 2020; Chen et al., 2021; Zheng et al., 2021). In this study, the statistical characteristics of the sample group were similar to those of the general population. All researchers using the National Health Insurance Research Database (NHIRD) or its data subsets are required to sign a written agreement stating that they do not intend to obtain information that may violate the privacy of the patients or care providers. All personal information was delinked and deidentified to protect the privacy of the parties involved. This study was approved by the Joint Institutional Review Board of Taipei Medical University (approval no. N201804043).

### Patient selection process and the definition of the primary endpoint

**FIGURE 1:**
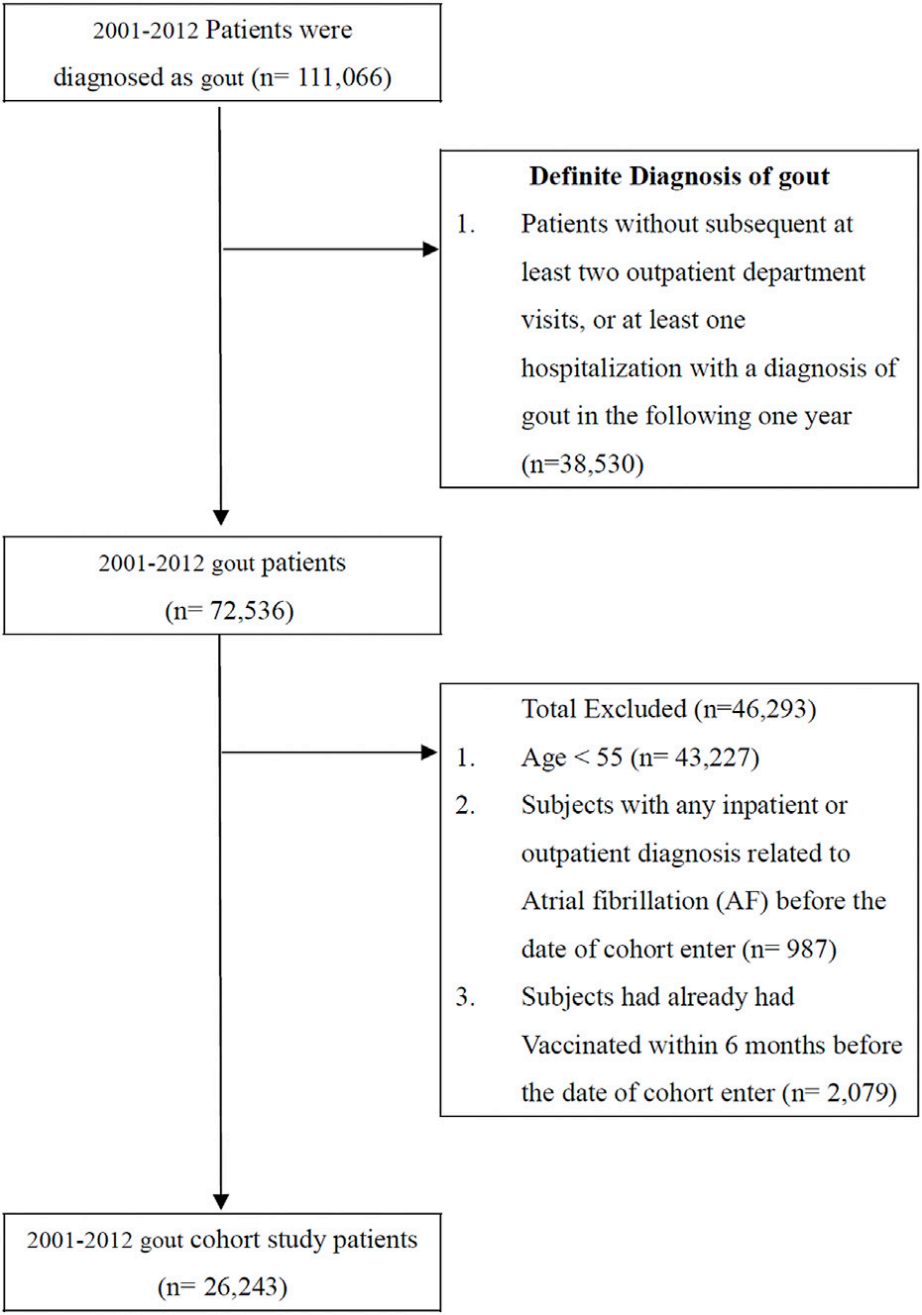
Data selection process.

Patients who were diagnosed with gout (International Classification of Diseases, Ninth Revision, Clinical Modification [ICD-9-CM] code 274. X) over a 12-year period (*n* = 111,066) from 1 January 2001, to 31 December 2012, were selected. However, patients without at least two subsequent outpatient department visits or at least one instance of hospitalization with a diagnosis of gout in the following year (*n* = 38,530) were excluded because of the uncertainty of gout diagnosis. The accuracy of the *ICD* diagnosis of gout has been validated in a previous study (Cheng et al., 2011). Another 46,293 patients meeting the following criteria were excluded from the study: patients aged 55 and younger (*n* = 43,227), patients with any inpatient or outpatient diagnosis related to AF before the date of enrollment in the cohort (*n* = 987), and patients who received influenza vaccination within 6 months before the enrollment date (*n* = 2,097). As a result, 26,243 patients were included in our final study cohort (Figure 1). In line with its public health policy, the government of Taiwan has been offering influenza vaccination free of charge since 1998 for citizens over 50 years of age with systemic diseases, such as chronic pulmonary disease, cardiovascular disease, chronic liver infection with cirrhosis, or type 2 diabetes mellitus, and since 2001 for all individuals over 65 years of age (Taiwan Centers for Disease Control, 2021). Here, we identified the history of vaccination with the *ICD-9-CM* code V048 and/or with the vaccine drug codes. The primary endpoint of our study was the occurrence of AF (*ICD-9-CM* code 427.31) in patients with gout during the influenza season (October to March), noninfluenza season (April to September), and all seasons over the follow-up years. All patients were followed up until they received a diagnosis of AF, dropped out from the NHI program, were lost to follow-up, died, or until 31 December 2012.

### Potential confounders

We selected the potential confounders of our cohort on the basis of sociodemographic characteristics (age, sex, urbanization level, and monthly income), history of inpatient and outpatient visits before study entry, comorbidities (Charlson Comorbidity Index [CCI], diabetes mellitus, hypertension, dyslipidemia, chronic obstructive pulmonary disease [COPD], cirrhosis, acute myocardial infarction [AMI], Ischemic heart disease, peripheral vascular disease), and medication use (allopurinol, benzbromarone, colchicine, aspirin, statins, renin–angiotensin–aldosterone system inhibitors [RAASIs], and metformin).

### Statistical analysis

First, we used a propensity score (PS) method to reduce the selection bias in the comparison between the vaccinated and unvaccinated groups by accounting for the covariates with a logistic regression model (Rosenbaum and Rubin, 1983; D'Agostino, 1998). We employed the chi-square test and *t*-test for categorical and continuous variables, respectively. We then analyzed the correlation between influenza vaccination and AF in patients with gout by using Cox proportional hazards regression analysis. We also examined the association between the seasonal effect of vaccination and the risk of AF. Subsequently, we determined the dose–response effect of influenza vaccination on the risk of AF. We divided patients with gout into four groups according to their vaccination status: unvaccinated patients, patients who received one vaccine dose, patients who received two or three vaccine doses, and patients who received four or more vaccine doses. These data were stratified by the age, sex, comorbidities, and medication use of the patients. Next, we used sensitivity analysis to evaluate the differences and similarities between influenza vaccination and the risk of AF in patients with gout. All statistical analyses were performed using IBM SPSS Statistics version 22.0 (IBM, Armonk, NY, United States) and SAS version 9.4 (SAS Institute, Cary, NC, United States). A *p*-value less than 0.05 was considered statistically significant.

## Results

### Baseline characteristics

A total of 26,243 eligible patients with gout were included in our cohort. Among these patients, 50.30% (13,201) were vaccinated against influenza, and the remaining 49.69% (13,042) were not. We observed a considerable difference in the age distribution, urbanization level, and monthly income between the two groups (Table 1). Compared with vaccinated patients, unvaccinated patients had higher times of inpatient and outpatient visits. In addition, compared with the vaccinated group, significantly higher prevalence of dyslipidemia and cirrhosis in the unvaccinated group was observed. The prevalence of hypertension, COPD and ischemic heart disease were significantly higher in vaccinated group. Compared with unvaccinated patients, those who were vaccinated demonstrated increased long-term use (≥28 days) of gout medications, such as allopurinol, benzbromarone, and colchicine. Moreover, those who were vaccinated demonstrated increased long-term use of aspirin, statins, RAASIs, and metformin. However, we observed no significant difference in the CCI between the vaccinated and unvaccinated groups.

**TABLE 1:** Characteristic of the sample population.

	Whole cohort (*n* = 26243)		Unvaccinated (*n* = 13201)		Vaccinated (*n* = 13042)		*p* ^a^
	***n***	**%**	***n***	**%**	**n**	**%**	
Age, years (Mean ± SD)	67.12 (8.47)		64.60 (8.60)		69.67 (7.53)	<0.001	
55–64	12317	46.93	8316	63.00	4001	30.68	<0.001
65–74	8863	33.77	2994	22.68	5869	45.00	
≥75	5063	19.29	1891	14.32	3172	24.32	
Gender							
Female	11161	42.53	5559	42.11	5602	42.95	0.167
Male	15082	57.47	7642	57.89	7440	57.05	
History of inpatient visits before study entry (Mean ± SD)	1.02 ± 2.89	1.14 ± 2.96	0.90 ± 2.81	<0.001			
History of outpatient visits before study entry (Mean ± SD)	136.17 ± 134.33	149.18 ± 141.64	122.99 ± 125.12	<0.001			
CCI							
0	7226	27.53	3729	28.25	3497	26.81	0.062
1	6676	25.44	3305	25.04	3371	25.85	
2	4945	18.84	2459	18.63	2486	19.06	
≥3	7396	28.18	3708	28.09	3688	28.28	
Comorbidities							
Diabetes	7627	29.06	3873	29.34	3754	28.78	0.322
Hypertension	16929	64.51	8120	61.51	8809	67.54	<0.001
Dyslipidemia	10188	38.82	5410	40.98	4778	36.64	<0.001
COPD	7701	29.34	3488	26.42	4213	32.30	<0.001
Cirrhosis	7443	28.36	3922	29.71	3521	27.00	<0.001
AMI	451	1.72	222	1.68	229	1.76	0.644
Ischemic heart disease	8805	33.55	4094	31.01	4711	36.12	<0.001
Peripheral vascular disease	2738	10.43	1314	9.95	1424	10.92	0.011
Allopurinol							
<28 days	19455	74.13	10425	78.97	9030	69.24	<0.001
≥28 days	6788	25.87	2776	21.03	4012	30.76	
Benzbro							
<28 days	13925	53.06	7741	58.64	6184	47.42	<0.001
≥28 days	12318	46.94	5460	41.36	6858	52.58	
Colchicine							
<28 days	11924	45.44	6196	46.94	5728	43.92	<0.001
≥28 days	14319	54.56	7005	53.06	7314	56.08	
Aspirin							
<28 days	13903	52.98	8238	62.40	5665	43.44	<0.001
≥28 days	12340	47.02	4963	37.60	7377	56.56	
Statin							
<28 days	15369	58.56	8169	61.88	7200	55.21	<0.001
≥28 days	10874	41.44	5032	38.12	5842	44.79	
RAASI							
<28 days	9962	37.96	6160	46.66	3802	29.15	<0.001
≥28 days	16281	62.04	7041	53.34	9240	70.85	
Metformin							
<28 days	19581	74.61	10148	76.87	9433	72.33	<0.001
≥28 days	6662	25.39	3053	23.13	3609	27.67	
Level of urbanization							
Urban	18096	68.96	9800	74.24	8296	63.61	<0.001
Suburban	5240	19.97	2339	17.72	2901	22.24	
Rural	2907	11.08	1062	8.04	1845	14.15	
Monthly income (NT$)							
0	2422	9.23	1009	7.64	1413	10.83	<0.001
1–21000	7494	28.56	3227	24.45	4267	32.72	
21000–33300	8284	31.57	3612	27.36	4672	35.82	
≥33301	8043	30.65	5353	40.55	2690	20.63	

a Comparison between Unvaccinated and Vaccinated.

CCI, Charlson Comorbidity index; RAASI, renin-angiotensin-aldosterone system inhibitor.

### The risk of AF in the vaccinated and unvaccinated groups

The hazard ratio of AF among all patients with gout (vaccinated and unvaccinated) is presented in Table 2. With the aforementioned potential confounders adjusted for, the risk of AF in the vaccinated group was significantly lower than in the unvaccinated group during all seasons (adjusted hazards ratio [aHR] = 0.55, 95% CI = 0.49–0.62, *p* < 0.001). Compared with the unvaccinated group, a decreased risk of AF was observed during the influenza and noninfluenza seasons among the vaccinated group (aHR = 0.59, 95% CI = 0.50–0.68, and aHR = 0.50, 95% CI = 0.42–0.63 during the influenza and noninfluenza seasons, respectively). Both male and female patients had a significantly decreased risk of AF after vaccination during all seasons. We also observed a significantly decreased risk of AF among patients aged 55–64, 65–74, and ≥75 during all seasons (Table 2).

**TABLE 2:** Risk of atrial fibrillation among unvaccinated and vaccinated in study cohort.

All group (*n* = 26243)	Unvaccinated (total follow-Up 71073.8 person-years)				Vaccinated (total follow-Up 104373.7 person-years)				Adjusted HR^†^ (95% C.I.)
	No. of patients with AF	Incidence rate (per 10^5^ person-years) (95%C.I.)			No. of patients with AF	Incidence rate (per 10^5^ person-years) (95%C.I.)			
**Whole cohort**									
Influenza season	325	457.3	(407.6,	507.0)	506	484.8	442.6	527.0	0.59(0.50, 0.68)***
Non-influenza season	223	313.8	(272.6,	354.9)	316	302.8	269.4	336.1	0.50(0.42, 0.63)***
All seasons	548	771.0	(706.5,	835.6)	822	787.6	733.7	841.4	0.55(0.49, 0.62)***
**Age, 55–64^a^**									
Influenza season	118	243.2	(199.3,	287.1)	88	237.6	(188.0,	287.2)	0.66(0.50, 0.88)**
Non-influenza season	71	146.3	(112.3,	180.4)	47	126.9	(90.6,	163.2)	0.63(0.43, 0.92)*
All season	189	389.5	(334.0,	445.0)	135	364.5	(303.0,	426.0)	0.65(0.52, 0.82)***
**Age, 65–74^b^**									
Influenza season	124	832.8	(686.2,	979.4)	245	521.1	(455.9,	586.4)	0.47(0.38, 0.59)***
Non-influenza season	87	584.3	(461.5,	707.1)	152	323.3	(271.9,	374.7)	0.40(0.31, 0.53)***
All season	211	1417.1	(1225.9,	1608.3)	397	844.5	(761.4,	927.5)	0.44(0.37, 0.53)***
**Age, ≥75^c^**									
Influenza season	83	1083.5	(850.4,	1316.6)	173	851.2	(724.3,	978.0)	0.65(0.50, 0.84)***
Non-influenza season	65	848.5	(642.3,	1054.8)	117	575.6	(471.3,	679.9)	0.57(0.42, 0.78)***
All season	148	1932.1	(1620.8,	2243.3)	290	1426.8	(1262.6,	1591.0)	0.62(0.50, 0.75)***
**Female^d^**									
Influenza season	115	389.8	(318.5,	461.0)	200	444.7	(383.1,	506.4)	0.62(0.49, 0.80)***
Non-influenza season	107	362.7	(294.0,	431.4)	132	293.5	(243.5,	343.6)	0.45(0.34, 0.58)***
All season	222	752.5	(653.5,	851.4)	332	738.3	(658.9,	817.7)	0.54(0.44, 0.64)***
**Male^e^**									
Influenza season	210	505.2	(436.8,	573.5)	306	515.1	(457.4,	572.8)	0.56(0.47, 0.68)***
Non-influenza season	116	279.0	(228.3,	329.8)	184	309.7	(265.0,	354.5)	0.56(0.43, 0.71)***
All season	326	784.2	(699.1,	869.3)	490	824.9	(751.8,	897.9)	0.56(0.48, 0.65)***

a Total follow-up 48523.5 person-year for unvaccinated and 37037.0 for Vaccinated.

b Total follow-up 14890.0 person-year for unvaccinated and 47011.5 for Vaccinated.

c Total follow-up 7660.2 person-year for unvaccinated and 20325.2 for Vaccinated.

d Total follow-up 29503.1 person-year for unvaccinated and 44970.0 for Vaccinated.

e Total follow-up 41570.7 person-year for unvaccinated and 59403.7 for Vaccinated.

† Main model is adjusted for age, sex, h**istory of inpatient visits before study entry,** history of outpatient visits before study entry, Charlson comorbidity index, diabetes, hypertension, dyslipidemia, **COPD, Cirrhosis, AMI, ischemic heart disease, peripheral vascular disease,** level of urbanization, Monthly income in propensity score.

C.I., confidence interval; HR, hazard ratio.

### Sensitivity analysis of the association between different times of vaccination and the risk of atrial fibrillation

Main model analysis revealed a significantly lower risk of AF after the administration of more than two vaccine doses during the influenza season (aHR = 0.85, 95% CI = 0.69–1.04; aHR = 0.72, 95% CI = 0.60–0.87; and aHR = 0.40, 95% CI = 0.33–0.49 for the first dose, second and third doses, and ≥fourth dose, respectively). With the covariates of medication use adjusted for (allopurinol, benzbromarone, colchicine, aspirin, statins, RAASIs, and metformin), the risk of AF significantly decreased after the administration of more than two vaccine doses. Among patients aged above 65, a significantly decreased risk of AF was observed after the administration of more than two vaccine doses (aHR = 0.79, 95% CI = 0.62–1.01; aHR = 0.70, 95% CI = 0.56–0.87; and aHR = 0.38, 95% CI = 0.31–0.47 for the first dose, second and third doses, and ≥fourth dose, respectively). After the administration of more than four vaccine doses, both male and female patients demonstrated a significantly decreased risk of AF. Moreover, among patients with or without diabetes, dyslipidemia, hypertension, COPD, cirrhosis, ischemic heart disease, and peripheral vascular disease, significantly lower risk of AF was observed after receiving more than four times of vaccination. In patients on allopurinol, benzbromarone, colchicine, aspirin, statins, RAASIs, or metformin (either short- or long-term use), the risk of AF significantly decreased with the administration of more vaccine doses (Table 3).

**TABLE 3:** Sensitivity analysis of adjusted HRs of vaccination in risk reduction of atrial fibrillation in influenza season.

	Unvaccinated	Vaccinated			*p* For trend
		1	2–3	≥4	
	Adjusted HR (95% C.I.)	Adjusted HR (95% C.I.)	Adjusted HR (95% C.I.)	Adjusted HR (95% C.I.)	
Main model^†^	1.00	0.85(0.69, 1.04)	0.72(0.60, 0.87)^***^	0.40(0.33, 0.49)^***^	<0.001
Additional covariates^‡^					
Main model + Allopurinol	1.00	0.84(0.69, 1.04)	0.72(0.59, 0.86)^***^	0.40(0.33, 0.48)^***^	<0.001
Main model + Benzbro	1.00	0.85(0.69, 1.05)	0.73(0.60, 0.88)^***^	0.41(0.34, 0.49)^***^	<0.001
Main model + Colchicine	1.00	0.85(0.69, 1.04)	0.72(0.60, 0.87)^***^	0.40(0.34, 0.49)^***^	<0.001
Main model + Aspirin	1.00	0.82(0.67, 1.01)	0.68(0.57, 0.83)^***^	0.38(0.31, 0.46)^***^	<0.001
Main model + Statin	1.00	0.85(0.69, 1.05)	0.73(0.61, 0.88)^***^	0.41(0.34, 0.50)^***^	<0.001
Main model + RAASI	1.00	0.83(0.67, 1.02)	0.70(0.58, 0.84)^***^	0.38(0.32, 0.46)^***^	<0.001
Main model + Metformin	1.00	0.85(0.69, 1.04)	0.72(0.60, 0.87)^***^	0.40(0.33, 0.49)^***^	<0.001
Subgroup effects					
Age, years					
55–64	1.00	0.85(0.57, 1.27)	0.70(0.47, 1.04)	0.49(0.32, 0.76)^**^	<0.001
≥65	1.00	0.79(0.62, 1.01)	0.70(0.56, 0.87)^***^	0.38(0.31, 0.47)^***^	<0.001
Sex					
Female	1.00	0.92(0.66, 1.28)	0.74(0.54, 1.00)	0.44(0.33, 0.60)^***^	<0.001
Male	1.00	0.80(0.62, 1.05)	0.71(0.56, 0.91)^**^	0.38(0.30, 0.48)^***^	<0.001
Diabetes					
No	1.00	0.83(0.65, 1.06)	0.71(0.57, 0.89)^**^	0.39(0.32, 0.49)^***^	<0.001
Yes	1.00	0.89(0.60, 1.33)	0.74(0.52, 1.07)	0.42(0.29, 0.61)^***^	<0.001
Dyslipidemia					
No	1.00	0.88(0.69, 1.13)	0.80(0.64, 1.00)^*^	0.46(0.37, 0.57)^***^	<0.001
Yes	1.00	0.78(0.54, 1.12)	0.57(0.40, 0.81)^**^	0.30(0.21, 0.42)^***^	<0.001
Hypertension					
No	1.00	0.82(0.56, 1.18)	0.65(0.46, 0.93)^*^	0.50(0.36, 0.70)^***^	<0.001
Yes	1.00	0.86(0.67, 1.10)	0.74(0.59, 0.92)^**^	0.36(0.28, 0.45)^***^	<0.001
COPD					
No	1.00	0.87(0.68, 1.11)	0.67(0.53, 0.85)^**^	0.42(0.34, 0.53)^***^	<0.001
Yes	1.00	0.81(0.56, 1.17)	0.79(0.58, 1.08)	0.36(0.26, 0.51)^***^	<0.001
Cirrhosis					
No	1.00	0.79(0.63, 1.01)	0.64(0.51, 0.79)^***^	0.39(0.31, 0.48)^***^	<0.001
Yes	1.00	1.06(0.69, 1.62)	1.07(0.73, 1.57)	0.46(0.31, 0.69)^***^	<0.001
AMI					
No	1.00	0.82(0.67, 1.02)	0.71(0.59, 0.86)^***^	0.40(0.33, 0.48)^***^	<0.001
Yes	1.00	2.85(0.74, 10.99)	1.68(0.40, 7.05)	1.17(0.27, 5.02)	0.999
Ischemic heart disease					
No	1.00	0.82(0.62, 1.08)	0.69(0.54, 0.89)^**^	0.47(0.37, 0.59)^***^	<0.001
Yes	1.00	0.86(0.63, 1.18)	0.73(0.55, 0.97)^*^	0.31(0.23, 0.42)^***^	<0.001
Peripheral vascular disease					
No	1.00	0.85(0.68, 1.05)	0.73(0.60, 0.89)^**^	0.40(0.33, 0.49)^***^	<0.001
Yes	1.00	0.81(0.39, 1.68)	0.61(0.32, 1.17)	0.41(0.22, 0.79)^**^	0.006
Allopurinol					
<28 days	1.00	0.77(0.59, 1.00)^*^	0.78(0.62, 0.99)^*^	0.36(0.28, 0.46)^***^	<0.001
≥28 days	1.00	0.98(0.70, 1.37)	0.61(0.44, 0.85)^**^	0.47(0.35, 0.63)^***^	<0.001
Benzbro					
<28 days	1.00	1.00(0.75, 1.33)	0.89(0.68, 1.15)	0.40(0.30, 0.53)^***^	<0.001
≥28 days	1.00	0.72(0.53, 0.97)^*^	0.60(0.46, 0.78)^***^	0.40(0.31, 0.52)^***^	<0.001
Colchicine					
<28 days	1.00	0.63(0.44, 0.89)^**^	0.69(0.51, 0.93)^*^	0.34(0.25, 0.46)^***^	<0.001
≥28 days	1.00	1.01(0.79, 1.31)	0.75(0.59, 0.96)^*^	0.45(0.36, 0.58)^***^	<0.001
Aspirin					
<28 days	1.00	0.95(0.66, 1.37)	0.67(0.47, 0.97)^*^	0.47(0.33, 0.67)^***^	<0.001
≥28 days	1.00	0.77(0.60, 0.99)^*^	0.68(0.54, 0.84)^***^	0.35(0.28, 0.44)^***^	<0.001
Statin					
<28 days	1.00	0.79(0.61, 1.03)	0.65(0.51, 0.83)^***^	0.43(0.34, 0.55)^***^	<0.001
≥28 days	1.00	0.96(0.69, 1.35)	0.87(0.65, 1.18)	0.39(0.29, 0.54)^***^	<0.001
RAASI					
<28 days	1.00	0.60(0.36, 0.99)^*^	0.81(0.55, 1.21)	0.36(0.23, 0.56)^***^	<0.001
≥28 days	1.00	0.88(0.70, 1.11)	0.67(0.54, 0.84)^***^	0.39(0.32, 0.48)^***^	<0.001
Metformin					
<28 days	1.00	0.84(0.67, 1.06)	0.67(0.54, 0.84)^***^	0.41(0.33, 0.51)^***^	<0.001
≥28 days	1.00	0.85(0.55, 1.31)	0.89(0.62, 1.29)	0.39(0.26, 0.57)^***^	<0.001

* *p*< 0.05.

** *p*< 0.01.

*** *p*< 0.001.

† Main model is adjusted for age, sex, history of inpatient visits before study entry, history of outpatient visits before study entry, Charlson comorbidity index, diabetes, hypertension, dyslipidemia, COPD, Cirrhosis, AMI, ischemic heart disease, peripheral vascular disease, level of urbanization, Monthly income in propensity score.

‡ The models were adjusted for covariates in the main model as well as each additional listed covariate.

C.I., confidence interval; HR, hazard ratio.

During both the noninfluenza season and all seasons, main model analysis indicated a significantly decreased risk of AF with the administration of more vaccine doses (aHR = 0.86, 95% CI = 0.68–1.10; aHR = 0.62, 95% CI = 0.49–0.79; and aHR = 0.30, 95% CI = 0.24–0.38 for the first dose, second and third doses, and ≥fourth dose, respectively, during the noninfluenza season, and aHR = 0.86, 95% CI = 0.73–1.00; aHR = 0.68, 95% CI = 0.59–0.79; and aHR = 0.36, 95% CI = 0.31–0.42 for the first dose, second and third doses, and ≥fourth dose, respectively, during all seasons). Subgroup analysis indicated that both male and female patients, patients above or below 65 years of age, patients with or without comorbidities, and patients on medications exhibited a significantly decreased risk of AF after the administration of more vaccine doses during both the noninfluenza season and all seasons (Tables 4, 5).

**TABLE 4:** Sensitivity analysis of adjusted HRs of vaccination in risk reduction of atrial fibrillation in non-influenza season.

	Unvaccinated	Vaccinated			*p* For trend
		1	2–3	≥4	
	Adjusted HR (95% C.I.)	Adjusted HR (95% C.I.)	Adjusted HR (95% C.I.)	Adjusted HR (95% C.I.)	
Main model^†^	1.00	0.86(0.68, 1.10)	0.62(0.49, 0.79)^***^	0.30(0.24, 0.38)^***^	<0.001
Additional covariates^‡^					
Main model + Allopurinol	1.00	0.86(0.67, 1.10)	0.62(0.49, 0.78)^***^	0.30(0.24, 0.38)^***^	<0.001
Main model + Benzbro	1.00	0.87(0.68, 1.11)	0.63(0.50, 0.79)^***^	0.31(0.24, 0.39)^***^	<0.001
Main model + Colchicine	1.00	0.87(0.68, 1.10)	0.62(0.49, 0.79)^***^	0.30(0.24, 0.39)^***^	<0.001
Main model + Aspirin	1.00	0.84(0.65, 1.07)	0.59(0.47, 0.75)^***^	0.28(0.22, 0.36)^***^	<0.001
Main model + Statin	1.00	0.87(0.68, 1.11)	0.63(0.50, 0.80)^***^	0.31(0.24, 0.40)^***^	<0.001
Main model + RAASI	1.00	0.85(0.67, 1.08)	0.61(0.48, 0.77)^***^	0.29(0.23, 0.37)^***^	<0.001
Main model + Metformin	1.00	0.86(0.68, 1.10)	0.62(0.49, 0.79)^***^	0.30(0.24, 0.39)^***^	<0.001
Subgroup effects					
Age, years					
55–64	1.00	1.08(0.68, 1.74)	0.54(0.30, 0.96)^*^	0.35(0.18, 0.69)^**^	<0.001
≥65	1.00	0.79(0.59, 1.04)	0.62(0.48, 0.81)^***^	0.30(0.23, 0.38)^***^	<0.001
Sex					
Female	1.00	0.66(0.45, 0.97)^*^	0.58(0.41, 0.82)^**^	0.29(0.20, 0.41)^***^	<0.001
Male	1.00	1.05(0.77, 1.45)	0.66(0.48, 0.90)^**^	0.32(0.23, 0.40)^***^	<0.001
Diabetes					
No	1.00	1.01(0.76, 1.35)	0.71(0.53, 0.93)^*^	0.35(0.27, 0.47)^***^	<0.001
Yes	1.00	0.60(0.38, 0.96)^*^	0.46(0.30, 0.71)^***^	0.20(0.13, 0.33)^***^	<0.001
Dyslipidemia					
No	1.00	0.90(0.67, 1.20)	0.65(0.49, 0.85)^**^	0.33(0.24, 0.43)^***^	<0.001
Yes	1.00	0.79(0.51, 1.23)	0.57(0.37, 0.88)^*^	0.25(0.16, 0.40)^***^	<0.001
Hypertension					
No	1.00	1.15(0.74, 1.78)	0.45(0.27, 0.77)^**^	0.40(0.25, 0.64)^***^	<0.001
Yes	1.00	0.76(0.57, 1.03)	0.66(0.51, 0.86)^**^	0.27(0.20, 0.35)^***^	<0.001
COPD					
No	1.00	0.81(0.59, 1.10)	0.64(0.47, 0.86)^**^	0.34(0.25, 0.46)^***^	<0.001
Yes	1.00	0.96(0.65, 1.41)	0.58(0.40, 0.85)^**^	0.25(0.17, 0.38)^***^	<0.001
Cirrhosis					
No	1.00	0.79(0.60, 1.05)	0.60(0.46, 0.77)^***^	0.27(0.21, 0.36)^***^	<0.001
Yes	1.00	1.16(0.70, 1.92)	0.73(0.44, 1.23)	0.44(0.27, 0.72)^***^	<0.001
AMI					
No	1.00	0.87(0.68, 1.11)	0.63(0.50, 0.79)^***^	0.31(0.24, 0.39)^***^	<0.001
Yes	1.00	0.66(0.13, 3.22)	0.21(0.04, 1.07)	-	0.057
Ischemic heart disease					
No	1.00	0.97(0.70, 1.34)	0.68(0.50, 0.93)^*^	0.34(0.25, 0.47)^***^	<0.001
Yes	1.00	0.73(0.50, 1.05)	0.53(0.38, 0.76)^***^	0.25(0.17, 0.36)^***^	<0.001
Peripheral vascular disease					
No	1.00	0.81(0.62, 1.06)	0.59(0.46, 0.76)^***^	0.30(0.23, 0.38)^***^	<0.001
Yes	1.00	1.18(0.64, 2.17)	0.72(0.40, 1.28)	0.32(0.17, 0.60)^***^	<0.001
Allopurinol					
<28 days	1.00	0.97(0.72, 1.30)	0.63(0.47, 0.85)^**^	0.32(0.24, 0.43)^***^	<0.001
≥28 days	1.00	0.68(0.45, 1.05)	0.59(0.41, 0.87)^**^	0.27(0.18, 0.41)^***^	<0.001
Benzbro					
<28 days	1.00	0.84(0.60, 1.17)	0.60(0.43, 0.83)^**^	0.27(0.19, 0.39)^***^	<0.001
≥28 days	1.00	0.90(0.64, 1.29)	0.67(0.48, 0.93)^*^	0.35(0.25, 0.48)^***^	<0.001
Colchicine					
<28 days	1.00	0.72(0.48, 1.08)	0.50(0.34, 0.74)^***^	0.30(0.21, 0.44)^***^	<0.001
≥28 days	1.00	0.97(0.71, 1.31)	0.71(0.53, 0.95)^*^	0.31(0.22, 0.42)^***^	<0.001
Aspirin					
<28 days	1.00	0.93(0.61, 1.43)	0.66(0.43, 1.01)	0.16(0.09, 0.29)^***^	<0.001
≥28 days	1.00	0.80(0.60, 1.08)	0.57(0.43, 0.76)^***^	0.32(0.25, 0.42)^***^	<0.001
Statin					
<28 days	1.00	0.98(0.73, 1.31)	0.68(0.51, 0.90)^**^	0.31(0.23, 0.42)^***^	<0.001
≥28 days	1.00	0.66(0.42, 1.03)	0.55(0.37, 0.82)^**^	0.31(0.21, 0.46)^***^	<0.001
RAASI					
<28 days	1.00	0.95(0.59, 1.53)	0.65(0.40, 1.05)	0.18(0.09, 0.35)^***^	<0.001
≥28 days	1.00	0.82(0.62, 1.09)	0.60(0.46, 0.78)^***^	0.32(0.25, 0.42)^***^	<0.001
Metformin					
<28 days	1.00	0.94(0.72, 1.24)	0.66(0.50, 0.86)^**^	0.31(0.24, 0.41)^***^	<0.001
≥28 days	1.00	0.59(0.33, 1.05)	0.53(0.32, 0.86)^*^	0.29(0.18, 0.47)^***^	<0.001

* *p*< 0.05.

** *p*< 0.01.

*** *p*< 0.001.

† Main model is adjusted for age, sex, history of inpatient visits before study entry, history of outpatient visits before study entry, Charlson comorbidity index, diabetes, hypertension, dyslipidemia, COPD, Cirrhosis, AMI, ischemic heart disease, peripheral vascular disease, level of urbanization, Monthly income in propensity score.

‡ The models were adjusted for covariates in the main model as well as each additional listed covariate.

C.I., confidence interval; HR, hazard ratio.

**TABLE 5:** Sensitivity analysis of adjusted HRs of vaccination in risk reduction of atrial fibrillation in all Sseasons.

	Unvaccinated	Vaccinated			*p* For trend
		1	2–3	≥4	
	Adjusted HR (95% C.I.)	Adjusted HR (95% C.I.)	Adjusted HR (95% C.I.)	Adjusted HR (95% C.I.)	
Main model^†^	1.00	0.86(0.73, 1.00)	0.68(0.59, 0.79)^***^	0.36(0.31, 0.42)^***^	<0.001
Additional covariates^‡^					
Main model + Allopurinol	1.00	0.85(0.73, 1.00)^*^	0.68(0.58, 0.78)^***^	0.36(0.31, 0.41)^***^	<0.001
Main model + Benzbro	1.00	0.86(0.73, 1.00)	0.69(0.59, 0.79)^***^	0.36(0.31, 0.42)^***^	<0.001
Main model + Colchicine	1.00	0.86(0.73, 1.00)	0.68(0.59, 0.79)^***^	0.36(0.31, 0.42)^***^	<0.001
Main model + Aspirin	1.00	0.83(0.71, 0.97)^*^	0.64(0.56, 0.75)^***^	0.34(0.29, 0.39)^***^	<0.001
Main model + Statin	1.00	0.86(0.74, 1.01)	0.69(0.60, 0.80)^***^	0.37(0.32, 0.43)^***^	<0.001
Main model + RAASI	1.00	0.84(0.71, 0.98)^*^	0.66(0.57, 0.76)^***^	0.35(0.30, 0.40)^***^	<0.001
Main model + Metformin	1.00	0.85(0.73, 1.00)^*^	0.68(0.59, 0.79)^***^	0.36(0.31, 0.42)^***^	<0.001
Subgroup effects					
Age, years					
55–64	1.00	0.94(0.69, 1.27)	0.64(0.46, 0.89)^**^	0.44(0.31, 0.64)^***^	<0.001
≥65	1.00	0.79(0.66, 0.95)^*^	0.67(0.57, 0.79)^***^	0.35(0.29, 0.41)^***^	<0.001
Sex					
Female	1.00	0.80(0.62, 1.02)	0.66(0.53, 0.83)^***^	0.37(0.29, 0.46)^***^	<0.001
Male	1.00	0.90(0.73, 1.10)	0.69(0.57, 0.84)^***^	0.36(0.29, 0.43)^***^	<0.001
Diabetes					
No	1.00	0.90(0.75, 1.08)	0.71(0.60, 0.84)^***^	0.38(0.32, 0.45)^***^	<0.001
Yes	1.00	0.75(0.56, 1.01)	0.60(0.46, 0.80)^***^	0.32(0.24, 0.42)^***^	<0.001
Dyslipidemia					
No	1.00	0.89(0.74, 1.08)	0.73(0.62, 0.87)^***^	0.40(0.34, 0.48)^***^	<0.001
Yes	1.00	0.79(0.59, 1.04)	0.57(0.43, 0.75)^***^	0.28(0.21, 0.37)^***^	<0.001
Hypertension					
No	1.00	0.94(0.71, 1.24)	0.58(0.43, 0.78)^***^	0.47(0.36, 0.61)^***^	<0.001
Yes	1.00	0.82(0.68, 0.99)^*^	0.70(0.59, 0.84)^***^	0.32(0.27, 0.38)^***^	<0.001
COPD					
No	1.00	0.84(0.70, 1.03)	0.66(0.55, 0.79)^***^	0.39(0.33, 0.47)^***^	<0.001
Yes	1.00	0.88(0.67, 1.15)	0.69(0.55, 0.88)^**^	0.31(0.24, 0.40)^***^	<0.001
Cirrhosis					
No	1.00	0.79(0.66, 0.95)^*^	0.62(0.53, 0.74)^***^	0.34(0.29, 0.40)^***^	<0.001
Yes	1.00	1.10(0.80, 1.52)	0.94(0.69, 1.27)	0.45(0.33, 0.61)^***^	<0.001
AMI					
No	1.00	0.84(0.72, 0.99)^*^	0.68(0.58, 0.78)^***^	0.36(0.31, 0.42)^***^	<0.001
Yes	1.00	1.43(0.54, 3.77)	0.90(0.32, 2.51)	0.43(0.13, 1.43)	0.166
Ischemic heart disease					
No	1.00	0.88(0.71, 1.08)	0.69(0.57, 0.84)^***^	0.42(0.35, 0.51)^***^	<0.001
Yes	1.00	0.80(0.63, 1.02)	0.64(0.51, 0.80)^***^	0.28(0.22, 0.35)^***^	<0.001
Peripheral vascular disease					
No	1.00	0.84(0.71, 0.99)^*^	0.68(0.58^***^	0.36(0.31, 0.42)^***^	<0.001
Yes	1.00	1.00(0.61, 1.63)	0.67(0.43, 1.03)	0.36(0.23, 0.57)^***^	<0.001
Allopurinol					
<28 days	1.00	0.85(0.70, 1.04)	0.72(0.60, 0.86)^***^	0.34(0.28, 0.41)^***^	<0.001
≥28 days	1.00	0.85(0.65, 1.11)	0.60(0.47, 0.78)^***^	0.38(0.30, 0.48)^***^	<0.001
Benzbro					
<28 days	1.00	0.93(0.75, 1.15)	0.76(0.62, 0.93)^**^	0.34(0.27, 0.43)^***^	<0.001
≥28 days	1.00	0.79(0.63, 0.99)^*^	0.62(0.51, 0.77)^***^	0.38(0.31, 0.46)^***^	<0.001
Colchicine					
<28 days	1.00	0.67(0.51, 0.87)**	0.61(0.48, 0.77)^***^	0.32(0.25, 0.41)^***^	<0.001
≥28 days	1.00	0.99(0.82, 1.21)	0.73(0.61, 0.88)^**^	0.39(0.32, 0.47)^***^	<0.001
Aspirin					
<28 days	1.00	0.94(0.71, 1.24)	0.67(0.50, 0.88)^**^	0.34(0.25, 0.45)^***^	<0.001
≥28 days	1.00	0.78(0.65, 0.95)^*^	0.63(0.53, 0.75)^***^	0.34(0.29, 0.40)^***^	<0.001
Statin					
<28 days	1.00	0.87(0.72, 1.06)	0.66(0.55, 0.80)^***^	0.38(0.31, 0.46)^***^	<0.001
≥28 days	1.00	0.83(0.64, 1.09)	0.74(0.58, 0.93)^*^	0.36(0.28, 0.46)^***^	<0.001
RAASI					
<28 days	1.00	0.75(0.54, 1.06)	0.74(0.54, 1.00)	0.28(0.19, 0.40)^***^	<0.001
≥28 days	1.00	0.86(0.72, 1.03)	0.64(0.54, 0.76)^***^	0.36(0.31, 0.43)^***^	<0.001
Metformin					
<28 days	1.00	0.88(0.74, 1.06)	0.67(0.56, 0.79)^***^	0.37(0.31, 0.44)^***^	<0.001
≥28 days	1.00	0.74(0.53, 1.04)	0.74(0.55, 0.99)^*^	0.34(0.25, 0.46)^***^	<0.001

* *p*< 0.05.

** *p*< 0.01.

*** *p*< 0.001.

† Main model is adjusted for age, sex, history of inpatient visits before study entry, history of outpatient visits before study entry, Charlson comorbidity index, diabetes, hypertension, dyslipidemia, COPD, Cirrhosis, AMI, ischemic heart disease, peripheral vascular disease, level of urbanization, Monthly income in propensity score.

‡ The models were adjusted for covariates in the main model as well as each additional listed covariate.

C.I, confidence interval; HR, hazard ratio.

## Discussion

### Major findings

In this section, we highlight the main findings of this population-based cohort study. *1*) In patients with gout, influenza vaccination was associated with a decreased risk of AF. *2*) In patients with or without chronic comorbidities, the risk of AF significantly decreased after influenza vaccination, and the potentially protective effect appeared to be dose-dependent. *3*) In patients above or below 65 years of age, the risk of AF significantly decreased after influenza vaccination. *4*) The potentially protective effect was observed during influenza season, noninfluenza season and all seasons.

### Mechanism of atrial fibrillation development in patients with gout

Both AF and gout have similar risk factors. According to the literature, the prevalence of AF in patients with gout is higher than in those without, although the mechanisms of both conditions remain unclear (Kim et al., 2016; Singh and Cleveland, 2018; Khan et al., 2021). Although the pathogenesis of AF remains only partially understood, the current evidence links AF to inflammation and autonomic nervous system complications. Several studies have demonstrated increased levels of inflammatory biomarkers (e.g., interleukin-2 [IL-2], IL-6, and IL-8, tumor necrosis factor-α, and C-reactive protein) in patients with AF (Engelmann and Svendsen, 2005; Liu et al., 2007; Guo et al., 2012). Other studies have also reported elevated levels of systemic inflammation markers (e.g., cytokines) in patients with gout, which may trigger oxidative stress, heart inflammation, and structural and electrical remodeling of the heart (Lee et al., 2007; Musa et al., 2013; Kuo et al., 2016b). Similar studies on African American patients have highlighted that an increase in the levels of serum uric acid is associated with an increased risk of AF, suggesting that uric acid is involved in atrial remodeling (Tamariz et al., 2011; KORANTZOPOULOS et al., 2012; Kuo et al., 2016a). All of these findings highlight the potential association between AF and gout.

### Mechanism of risk reduction of atrial fibrillation after influenza vaccination

According to the literature, influenza vaccination can reduce the risks of AF. In patients with influenza, inflammatory cytokines are produced and the sympathetic nervous system is activated (Chang et al., 2016). Infection with influenza may also cause myocarditis, which leads to AF by affecting the electrical system of the heart (Karjalainen et al., 1980; Rothberg et al., 2008). Therefore, influenza vaccination can prevent AF by reducing the aforementioned risks during an infection with influenza. In this study, we observed a robust protective effect for influenza vaccination. This may be because patients with gout are already in a state of inflammation (with elevated levels of inflammatory factors) and are susceptible to infection (Spaetgens et al., 2017). We also observed that this protective effect was further enhanced after influenza vaccination in patients with comorbidities. Furthermore, we observed that patients aged 65 and above with gout benefited more from influenza vaccination than patients aged 55–64 regardless of the season. This may be because the immune systems of older individuals are less capable of fighting off infections.

### Mechanism between the vaccination dose and atrial fibrillation risk

A dose effect was found in our study; patients with more vaccinations were associated with lower hazard ratio of AF. In previous study, significantly lower risk of AF was observed among heart failure patients who received greater numbers of influenza vaccination (Modin et al., 2019). The function of the memory immune cells in patients with chronic diseases are more likely to diminish over time than in normal people (Sester et al., 2013). Therefore, with more vaccinations, the protective effect might became greater. However, the present study also demonstrated that the potential dose effect was also observed during noninfluenza season. In the study from (Zhu et al 2009), significantly lower risk of venous thromboembolism event was observed across the 12 months evenly after influenza vaccination. A recent study from Pang et al. showed that the risk of in-hospital death was significantly lower in patients received influenza vaccination during both influenza and summer months (Pang et al., 2022). Therefore, the possible mechanism other than influenza infection prevention might exist. Anti-inflammatory and plaque stabilization after influenza vaccination are the potential mechanisms of decreasing risk of AF after vaccination among patients with gout(Bermúdez-Fajardo and Oviedo-Orta, 2011; Pothineni et al., 2017; Atoui et al., 2021). However, future study is warranted to validate the result of present study.

### Limitations

This study has several limitations. First, some parameters (e.g., body mass index), personal information (e.g., smoking status and alcohol consumption), and biochemical data (e.g., level of uric acid) were not available from the NHIRD. Therefore, we used a PS method to minimize bias. Second, because of the influenza vaccination policy of Taiwan, we excluded patients younger than 55 years. Therefore, future studies should include younger patients with gout. Third, this study had a retrospective and observational design. Therefore, prospective studies are required to validate our findings. Fourth, although previous study demonstrated the anti-inflammatory effect and plaque stabilization from influenza vaccination beyond the infection prevention effect (Bermúdez-Fajardo and Oviedo-Orta, 2011; Pothineni et al., 2017; Atoui et al., 2021), these findings still could not fully explain the finding of potential protective effect from vaccination across influenza season, noninfluenza season and all seasons in the present study. Potential unknown confounding factor could exist in the present study and future research is warranted to validate this finding. Fifth, healthy user bias potentially exist in this observational study (Shrank et al., 2011). In Taiwan, the influenza vaccine was provided free of charge to the patients with chronic disease. In addition, to minimize the bias, we further performed PS and adjusted age, sex, urbanization level, monthly incomes, comorbidities, inpatient and outpatient visits among vaccinated and unvaccinated patients in calculation of hazard ratio of AF risk. (Rosenbaum and Rubin, 1983; D'Agostino, 1998). Sixth, the selected cohort in the present study was during 2001–2012. Future study analysis from more recent data to validate the findings of present study is warranted.

## Conclusion

Influenza vaccination is associated with a decreased the risk of AF in patients with gout. This potentially protective effect seems to be dose-dependent.

## Data Availability

The data supporting the findings of this research were sourced from NHIRD in Taiwan. Owing to the legal restrictions imposed by the Government of Taiwan related to the Personal Information Protection Act, the database cannot be made publicly available.
